# Utilization of Treated Agricultural Residue Ash as Sodium Silicate in Alkali Activated Slag Systems

**DOI:** 10.3390/ma14020329

**Published:** 2021-01-11

**Authors:** Feraidon F. Ataie

**Affiliations:** Concrete Industry Management Program, California State University-Chico, 400 West First Street, Chico, CA 95926, USA; fataie@csuchico.edu

**Keywords:** alkali activated slag, silica fume, rice husk ash, rice straw ash, sodium silicate

## Abstract

This study investigated the influence of rice straw ash (RSA), rice husk ash (RHA), and silica fume (SF) on alkali activated slag (AAS) systems. RSA, RHA, and SF were treated with sodium hydroxide to improve their reactivity in AAS systems. Although addition of SF in AAS systems increased compressive strength, samples containing RSA or RHA had higher compressive strength than those having SF. Treated RSA or RHA further increased compressive strength of AAS samples. It was shown that samples containing treated ash samples had similar compressive strength to those made with sodium silica activator. Therefore, it is suggested that treated ash samples could be used as alternative sources of silica for AAS. Drying shrinkage of AAS samples increased considerably when treated RSA or RHA were used as partial replacement of slag. This could be attributed to higher silica modulus (SiO_2_/Na_2_O) ratio of samples containing treated ash, which in turn would lead to a finer pore size structure compared to control samples. However, SF significantly reduced drying shrinkage of AAS. This could be because SF reduces the permeability and porosity of AAS samples.

## 1. Introduction

Concrete is the most used substance in the world after water [1]. The production of portland cement, which is an essential ingredient of concrete, is an energy intensive process. This process is responsible for approximately 8% of the global carbon dioxide (CO_2_) emission [2,3]. Most of this CO_2_ emission comes from calcination of limestone during the portland cement production process [1,2].

Several possible methods, techniques, and materials have been suggested to mitigate the environmental impact of concrete production [1,4]. Increasing the efficiency of cement production process, improving the efficiency of cement in concrete, facilitating the partial replacement of cement in concrete, increasing concrete durability, and using alternative cementitious materials are among the plausible strategies to reduce the carbon footprint of concrete [1,4]. Alkali activated binders, also called alternative cementitious materials, are believed to be viable cementing materials for many concrete applications [2,5]. Granulated Blast Furnace Slag (slag hereinafter) was first used in 1940 to develop alkali activated binders [6]. Alkali activated binders are prepared by mixing an alkaline solution, such as sodium hydroxide (NaOH), with a solid alumino-silicate material, such as slag. The alkaline solution, which is referred to as the activator, is responsible for the cementing reaction of alkali activated systems. It has been shown that alkali activated materials, particularly alkali activated slag, could be produced with a comparable performance to that of the conventional concrete [7]. Studies have also revealed that alkali activated materials could be used as repair materials [8,9].

It is believed that alkali activated binders have lower environmental impact than that of the portland cement [2,5,7,10]. However, the environmental impact of alkali activated materials depends on several factors. The type and production process of alumino-silicate materials as well as type of activators are some of the influential factors on the environmental impact of alkali activated binders [2,5,10]. 

The performance of alkali activated binders is influenced by several factors such as Na_2_O content and SiO_2_/Na_2_O ratio (silicate modulus) of the activator solution [11,12,13,14]. For a given Na_2_O content, a higher silicate modulus increases compressive strength and autogenous shrinkage of alkali activated systems. Similarly, for a given SiO_2_/Na_2_O ratio, an increase in Na_2_O content improves compressive strength. An increase in Na_2_O results an increase in sodium hydroxide concentration; and an increase in silicate modulus leads to a higher silica content of the activator solution. Both of these factors, an increase in SiO_2_/Na_2_O ratio or in Na_2_O content, lead to a higher negative environmental impact of alkali activated binders, especially if sodium silicate is used as a silica source in activator solutions. Researchers have studied the usage of environmentally friendly materials to improve alkali activated binders’ properties. 

Zhang et al. [15] showed that partial replacement of fly ash with waste glass powder in alkali activated systems improves compressive strength. Similarly, sugar cane straw ash (SCSA) has been shown to increase the compressive strength of alkali activated slag when added as a partial replacement of slag [16]. Furthermore, another study dissolved waste glass in activator solutions to improve alkali activated slag properties [17]. Mejia et al. [18] used rice husk ash (RHA) as an alternative source of silica in activator solutions. However, they found that for a given SiO_2_/Na_2_O ratio sodium silicate showed superiority over RHA. 

This study investigated the impact of Rice Straw Ash (RSA), Rice Husk Ash (RHA), and silica fume (SF) on the compressive strength, heat of hydration, and drying shrinkage of alkali activated slag systems. Both treated and untreated SF, RSA, and RHA were used. The performance of systems containing these materials was compared with systems prepared with sodium silicate solutions. The objective of this research was to determine whether RSA, RHA, and SF could be used as effective sources of silica for alkali activated systems.

## 2. Materials and Methods

### 2.1. Materials

The chemical compositions of grade 120 slag, RSA, RHA, and SF used in this study are shown in Table 1. The chemical compositions of materials were obtained using X-ray fluorescence. ASTM standard graded sand was used for preparing mortar cubes [19]. ASC grade NaOH was used to make sodium hydroxide activator solution. Technical grade sodium silicate (28% silica, 9% sodium oxide, 63% water) was used.

### 2.2. Methods

**Figure 1:**
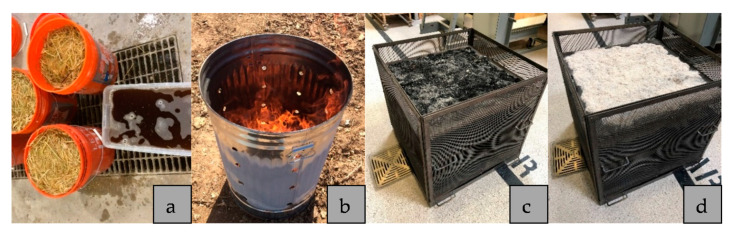
Rice husk ash (RHA) and rice straw ash (RSA) production process; (**a**) Water pretreatment; (**b**) initial burning; (**c**) ash after initial burning; (**d**) ash after final burning in the kiln.

Rice husks and rice straw were sourced locally. Rice hulls and rice straw pretreated with water prior to burning in order to produce ash with high amorphous silica and low loss on ignition [20,21]. Water pretreatment was done by soaking 1360 g of rice hulls or 907 g of rice straw in a 19-litter (5-gallon) bucket for 24 h. The bucket was filled with tap water. After 24 h, the biomass (rice hulls or rice straw) was rinsed out twice. The biomass was then dried in an oven at 100 °C. The dried rice hulls and rice straw were initially burned in a steel container. The resulted ash (with high loss of ignition) was then burned at 600 °C for one hour in a gas-fired pottery kiln to produce the final ash. This ash was then grinded in a laboratory ball mill to produce powder RHA and RSA. To grind the ash, 80 g of the ash was placed in a one-litter ball mill with twenty-five 1 cm grinding media; the ash was ground for one hour. Figure 1 depicts the production process of RHA and RSA. The chemical composition of RSA and RHA is given in Table 1. 

Sodium hydroxide activator solutions were prepared at three different molarities of 2 M, 4 M, and 6 M. Mortar samples were prepared for determining compressive strength, flow, and drying shrinkage. A liquid/solid (activator/cementitious materials) ratio of 0.7 (by mass) and a sand/cementitious material ratio of 2.75 were used for preparing mortar mixtures. A liquid/solid ratio of 0.6 were used for paste samples. This high activator (liquid) content was used to ensure samples containing RHA and RSA addition were workable. Mortar cubes were prepared and tested in accordance with ASTM C305 and C109 [22], respectively. Flow of mortar samples was determined following ASTM C1437 procedure [23]. To prepare paste samples, the activator and solid material were mixed at 600 rpm for 2 min followed by a one-minute rest, and then one minute of final mixing. An overhang mixer was used to mix paste samples. 

The drying shrinkage of mortar were measured according to ASTM C596 [24]; a deviation from the standard procedure was that samples were moist cured (instead of saturated lime water) in a curing room for two days before being subjected to drying. Heat of hydration of paste samples were measured using a four-channel isothermal calorimeter (Calmetrix, Boston, MA, USA). Approximately 35 g of paste were used for each measurement. Mortar and paste samples with and without silica fume, RHA, or RSA were prepared. SF, RHA, and RSA were added at three different replacement levels of 5%, 10%, and 15% of slag mass. 

**Figure 2:**
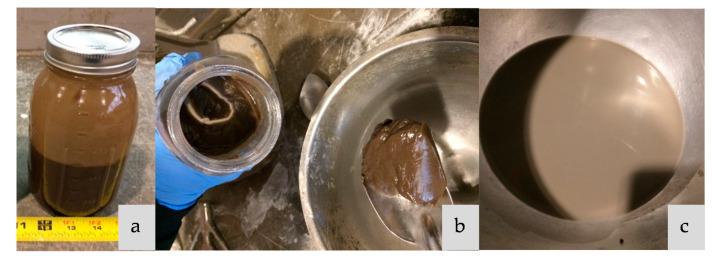
Ash treatment process (this process was used for SF-T, RSA-T, and RHA-T); (**a**) ash being soaked in NaOH; (**b**) ash after 24 h soaking; (**c**) dispersed treated ash in the activator solution.

Both treated and untreated SF, RSA and RHA were used. Untreated RSA or RHA refers to RSA or RHA being added to the mix without any further process after grinding. To treat RSA, RHA, or SF, the material was immersed in the activator solution for 24 h before being mixed with slag. The amount of activator used to soak the ash was three times the mass of the material (RSA, RHA, or SF) needed for a mixture; this amount was deducted from the total amount of activator of the mix to keep the liquid/solid ratio the same for all mixtures. Samples treated by this method are named RSA-T, RHA-T, or SF-T. Figure 2 shows the treatment process. Sodium silicate (Na-Si) was used to prepare activator solutions with SiO_2_/Na_2_O ratios of 0.29 and 0.58 for comparison with solutions made with treated ash or silica fume.

Table 2 shows modulus ratios sodium oxide contents of activator solutions prepared by using 4 M sodium hydroxide. It should be noted that SiO_2_/Na_2_O ratios and Na_2_O contents shown is based on assumption that 100% of silica content of treated materials would dissolve in sodium hydroxide activator.

## 3. Results and Discussion

### 3.1. Impact of RSA, RHA, and SF on Hydration Kinetics

**Figure 3:**
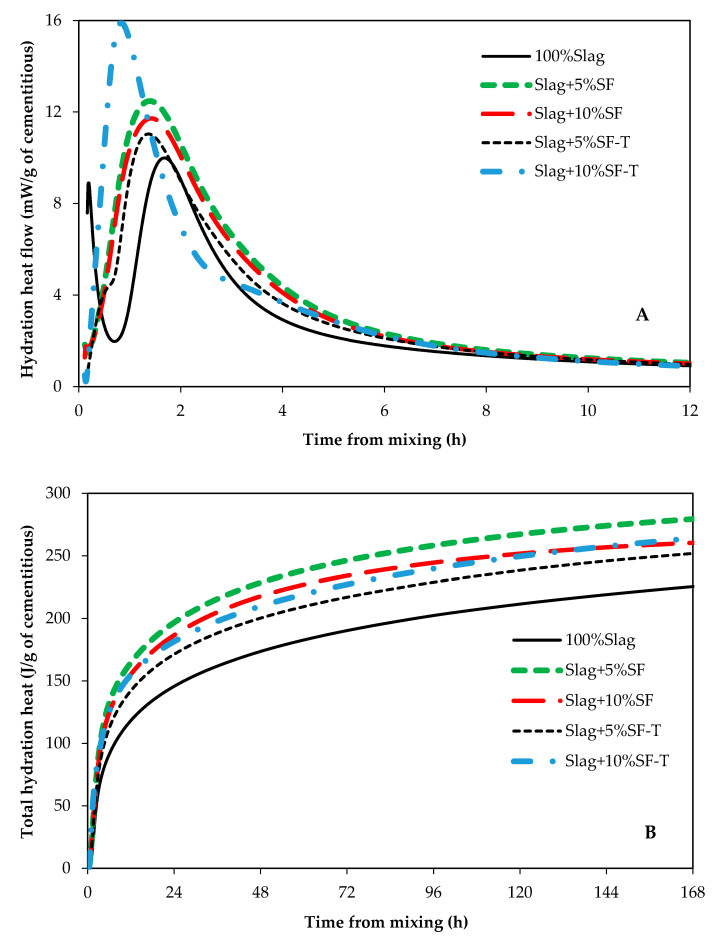
Hydration heat of samples containing silica fume. (**A**) heat flow, and (**B**) total hydration heat.

**Figure 4:**
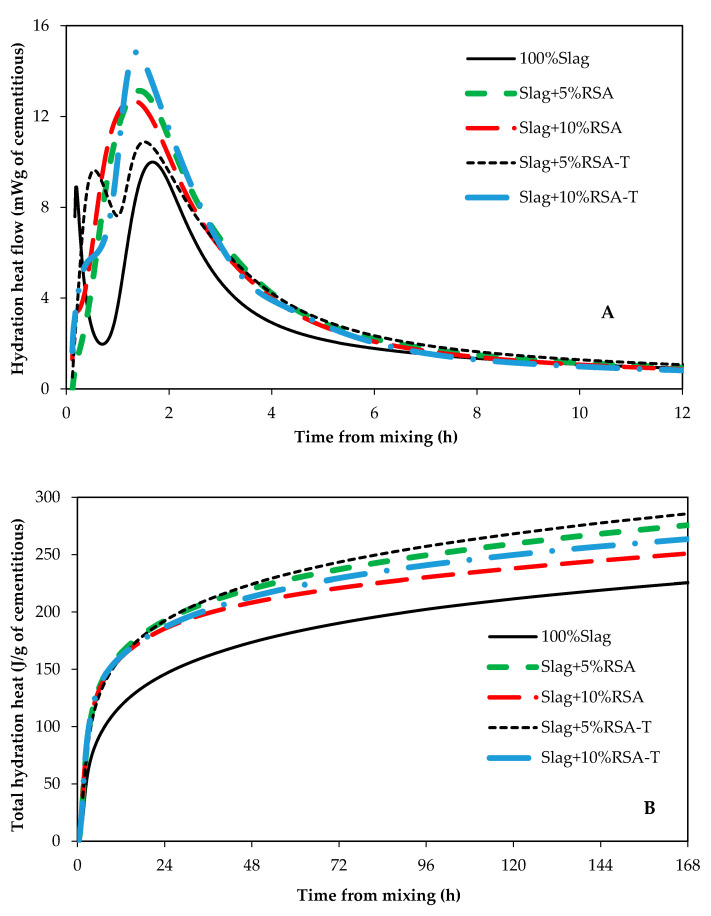
Hydration heat of samples containing rice straw ash. (**A**) heat flow, and (**B**) total hydration heat.

**Figure 5:**
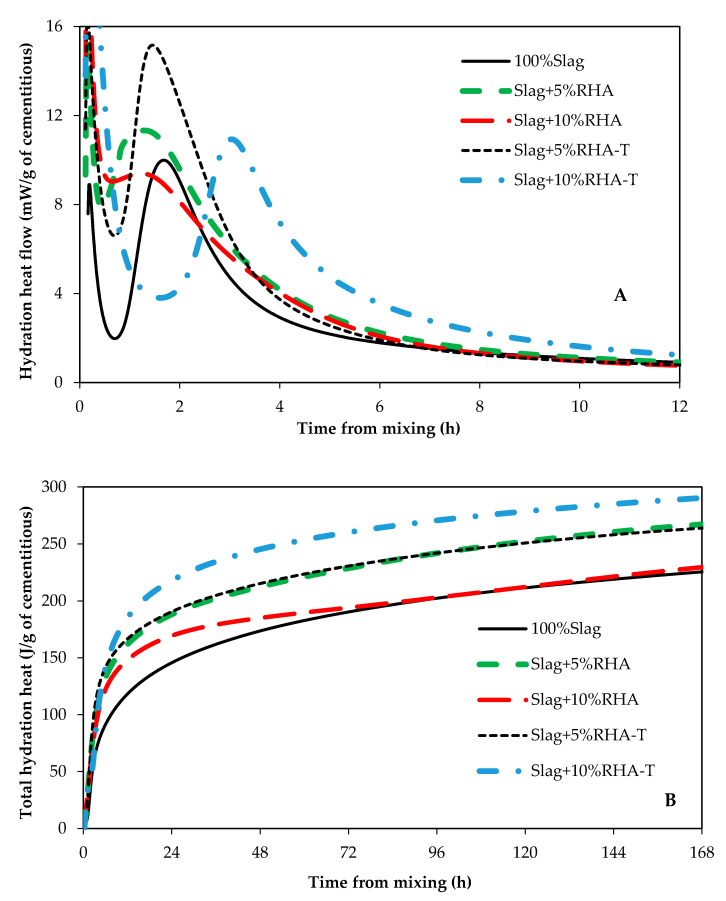
Hydration heat of samples containing rice hulls ash. (**A**) heat flow, and (**B**) total hydration heat.

It has been shown that the main hydration product of alkali activated slag is calcium silicate hydrate which incorporates a significant amount of aluminum (C(-A)-S-H) [6,25,26]. The heat of hydration of paste samples were measured to investigate the impact of RSA, RHA, and SF on hydration kinetics of alkali activated slag. The heat of hydration graphs for samples containing SF, RSA, and RHA are shown in Figure 3, Figure 4 and Figure 5, respectively. Control samples (100% Slag) showed a similar hydration graph commonly seen for portland cement such that induction, acceleration, and deceleration periods are all visible and distinguishable. However, samples containing either SF, SF-T, RSA, or RSA-T did not show any induction period. This could be attributed to the ability of SF and RSA to provide nucleation sites for C(-A)-S-H formation; also, dissolution of silicon ions from SF and RSA into the pore solution could also speed up formation and growth of hydration products. These factors could eliminate the induction period. Therefore, adding SF or RSA, particularly when they are treated, in alkali activated slag systems could accelerate the set time and early strength gain.

All samples containing either SF, RSA, or RHA showed higher heat of hydration compared to the control sample. When comparing samples that contained SF, it can be seen from Figure 3B that sample with 5%SF had the highest heat of hydration. The sample containing 5% treated RSA (Slag + 5%RSA-T) had the highest heat of hydration among samples that contained RSA (Figure 4B). However, comparing samples containing RHA, Figure 5B shows that Slag + 10%RHA-T had the highest amount of hydration heat. Samples containing SF showed almost no induction period (Figure 3A). A similar trend was seen in samples containing RSA (Figure 4A). It seems like the heat of hydration of AAS samples containing treated samples does not correlate to the compressive strength for the highest compressive strength was achieved in samples that contained 10% of SF-T, or RSA-T, or RHA-T. This could be because treated samples were immersed in the activator solutions for 24 h; during this stage, some heat could have been released by the materials. Therefore, the amount of hydration heat of samples containing treated samples would be lower than those containing untreated ones.

### 3.2. Impact of RSA, RHA, and SF on Mortar Flow 

The influence of RSA, RHA, and SF on mortar workability was investigated with the 4M activator solution. The addition of RSA, RHA, and SF as partial replacement of slag had variable effects on alkali activated slag mortar flow. The samples containing RSA or RHA decreased the flow, as shown in Table 3. However, the samples containing 5%SF or 5%RHA-T had a higher flow compared to the control (slag) sample. The lowest flow was recorded for samples containing 10%RHA-T while sample containing 5%SF had the highest flow. It has been shown that SF when used up to 15% in ultra-high performance concrete increases workability [27,28]. This has been attributed to lubrication effect of silica fume. Higher flowability of samples containing 5% SF or 5%RHA-T could be the lubrication effect of SF and RHA-T. However, the decrease in flow of samples containing RSA, RHA, RSA-T, or SF-T could be attributed to a couple of factors: (1) the absorption of activator solution by these materials due to their high surface areas [20,27]; (2) the faster formation and growth of hydration products in these samples.

### 3.3. Impact of RSA, RHA, and SF on Compressive Strength

**Figure 6:**
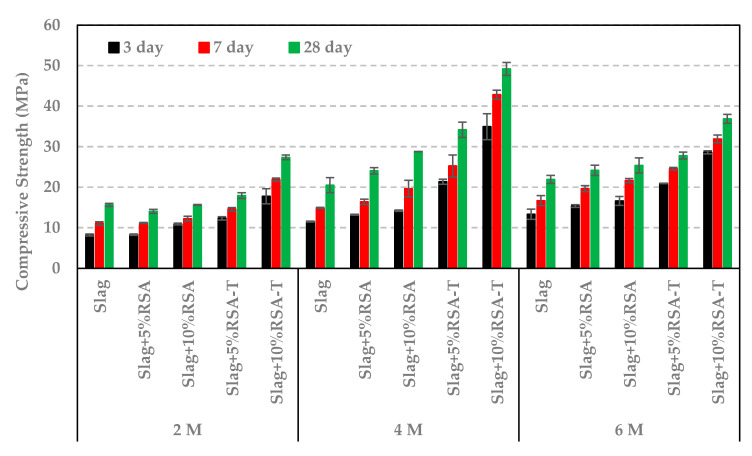
Compressive strength of mortar cubes at different activator concentrations.

The impact of activator concentration on compressive strength of alkali activated slag mortar samples was measured in order to obtain the optimum activator concentration (molarity). Activator solutions at three different concentrations (2 M, 4 M, and 6 M) were used. As shown in Figure 6, the compressive strength of control sample increased with an increase in the activator concentration. However, samples containing RSA or RSA-T performed best at 4M concentration. A decrease in the activator concentration increases the modulus ratio (SiO_2_/Na_2_O) and a decrease in Na_2_O % of the solution. Therefore, 4 M NaOH activator gives the optimum modulus ratio and sodium oxide content for samples containing RSA (treated or untreated). 

**Figure 7:**
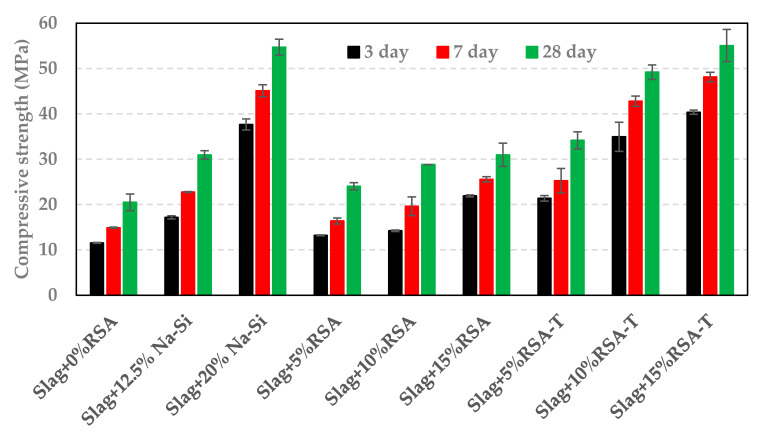
Comparison between RSA and sodium silicate.

The performance of samples containing RSA and RSA-T were compared to those containing sodium silicate (Na-Si). Sodium Silicate was added at 12.5% and 20% replacement of 4 M NaOH solution to prepare activator solutions with SiO_2_/Na_2_O ratios of 0.29 and 0.58, respectively. The samples containing sodium silicate (Slag + 12.5% Na-Si and Slag + 20%Na-Si) had higher compressive strength that those containing RSA, as shown in Figure 7. However, sample containing 5%RSA-T showed a higher compressive strength than the sample prepared with 12.5%Na-Si. Compressive strength of Slag + 20%Na-Si was higher than Slag + 10%RSA-T. However, the compressive strength of samples containing 15%RSA-T was slightly higher than the one made with 20%Na-Si. Figure 7 also shows that sample containing 15%RSA had similar compressive strength to the one made with 12.5%Na-Si. 

**Figure 8:**
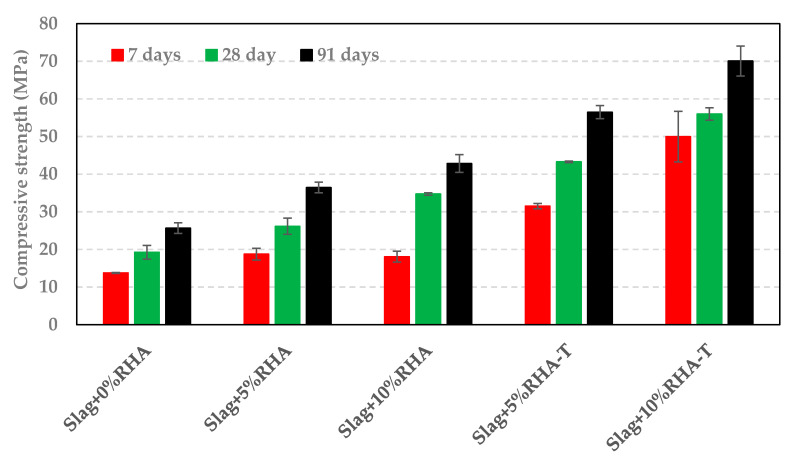
Compressive strength of samples containing RHA.

**Figure 9:**
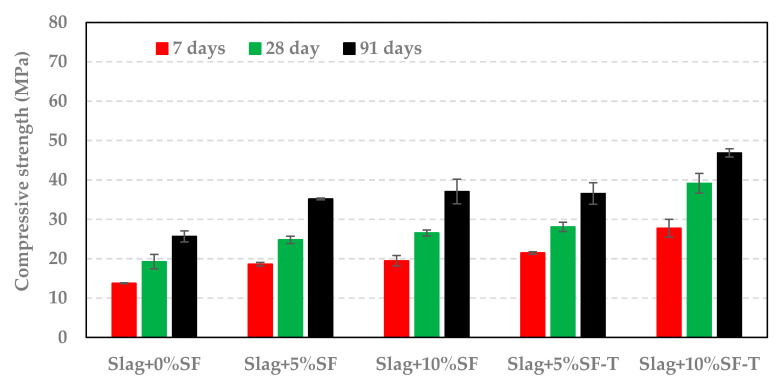
Compressive strength of samples containing silica fume.

The compressive strength of samples containing RHA or SF is shown in Figure 8 and Figure 9, respectively. As it can be seen in Figure 8, the compressive strength increased with an increase in RHA content. The compressive strength of samples containing treated RHA (RHA-T) was 50% higher than of those containing RHA (untreated). Furthermore, the samples containing 10%RHA had similar strength to the one made with 20%Na-Si. A comparison of results in Figure 7 with those presented in Figure 8 shows that the compressive strength of samples containing RHA or RHA-T is slightly higher than those containing RSA or RSA-T. This can be attributed to the higher silica content of RHA compared to RSA (see Table 1). Nevertheless, treated RSA or RHA (RSA-T or RHA-T) significantly increased the compressive strength compared to untreated ash samples.

For a given replacement level of slag, samples containing SF showed a similar compressive strength to those having RSA or RHA. However, samples containing SF-T had a much lower strength compared to those made with RSA-T or with RHA-T. Nevertheless, treating SF increased the reactivity of SF as samples containing SF-T had a higher compressive strength that those containing SF, as indicated in Figure 9.

It has been shown that the main hydration product of alkali activated slag is calcium silicate hydrate which incorporates a significant amount of aluminum (C(-A)-S-H) [6,25,26]. In slag systems activated with NaOH, C(-A)-S-H forms around slag grains [26,29]. This is proposed to suppress the dissolution of slag which slows the formation of hydration products down. In contrast, in slag systems activated with sodium silicate hydration products do not form on slag grains surfaces [26,29]. This promotes slag dissolution and hydration products formation, which leads to a higher compressive strength [13]. 

The addition of RSA, RHA, or SF in alkali activated slag could have a couple of effects on the hydration process. These materials could supplement the amount of reactive silica in the system which would lead to a higher amount of silicon ions in the pore solution; this also increases the silica modulus of the solution. On the other hand, RSA, RHA, and SF could provide nucleation sites for C(-A)-S-H and other hydration products formation which will in turn enhance the dissolution of slag. Both mechanisms promote hydration process and increase compressive strength. The higher compressive strength of samples containing RSA, RHA, or SF, as shown in Figure 7, Figure 8 and Figure 9, compared to control samples (slag only) could be due to the two aforementioned mechanisms. Similarly, it can be suggested that treating RSA, RHA, or SF with NaOH would dissolve silicon ions. Therefore, samples containing RSA-T, RHA-T, or SF-T have higher initial silicon content than those containing untreated materials; this would lead to higher initial silica moduli. Besides, RSA-T, RHA-T, or SF-T would provide more nucleation sites for hydration products. Consequently, samples containing RSA-T, RHA-T, or SF-T have higher compressive strengths than those containing untreated materials. 

It has been suggested that silica content of RHA dissolves in high pH solutions [18]. If it is assumed that all the silica of RSA or RHA dissolve as a result of treatment, samples containing 5% of RSA-T (5% RHA-T) and samples containing 10% of RSA-T (10%RHA-T) would have a silica modulus of 0.29 and 0.58, respectively. The samples containing SF-T would have slightly higher silica moduli. Samples prepared with 12.5%Na-Si and 20%Na-Si had a silica modulus of 0.29 and 0.58, respectively. As presented in Figure 7 and Figure 8, samples containing 5% RSA-T or 5%RHA-T outperformed the sample containing 12.5%Na-Si; this suggests that RSA-T or RHA-T could be used as sodium silicate in alkali activated systems. As it was mentioned earlier, for a given age and replacement level, samples containing SF-T had lower compressive strength than those containing RSA-T or RHA-T. This could be because RSA and RHA have higher internal surface areas than silica fume [20]. This could reduce the amount of silicon ions dissolved from the silica fume. Lower surface area would also mean that there are fewer number of nucleation sites in the system. Both factors could contribute to the compressive strength reduction.

### 3.4. Impact of RSA, RHA, and SF on Drying Shrinkage

**Figure 10:**
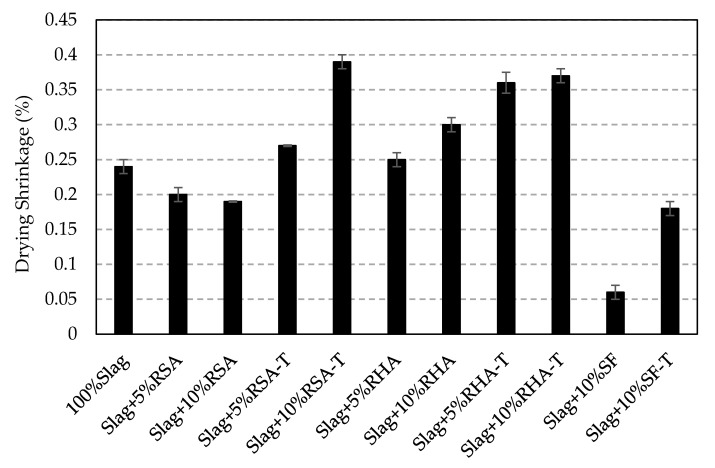
Drying shrinkage of AA mortar samples.

The drying shrinkage of AAS mortar samples is shown in Figure 10. Addition of RSA in mixtures reduced the drying shrinkage; however, samples containing RSA-T had a higher drying shrinkage compared to the control samples. Adding RHA or RHA-T to AAS mixtures increased the drying shrinkage. However, the addition of either SF or SF-T substantially reduced the drying shrinkage. Among all samples prepared, those that contained 10% treated ash samples (10%RSA-T or 10%RHA-T) showed the highest drying shrinkage (about 60% more than the control sample). The lowest drying shrinkage was obtained for sample containing 5% SF (75% reduction in drying shrinkage). 

It has been shown that alkali activated slag has a much higher drying shrinkage than portland cement [7,30,31]. This has been attributed to the finer pore structure and lower stiffness of AAS compared to that of portland cement systems [30,32]. Li et al. [33] suggested that a high pore pressure resulted due the fine pore structure of AAS leads to higher shrinkage of AAS samples. It has also been shown that an increase in the silica modulus will increase the pore structure fineness that leads to a higher drying shrinkage of AAS [31,34]. The increase in drying shrinkage of samples containing RHA and treated ash samples could be attributed to the higher silica modulus in these samples as ash samples contain a high amorphous silica content. It could be also possible that addition of ash samples further reduces the stiffness of the AAS pore structure due to the higher silica modulus. Although silica fume also contains a very high amount of amorphous silica, it did not increase drying shrinkage when added in AAS samples. This could be attributed to the fact that SF reduces permeability and porosity of AAS samples when added as partial replacement of slag [35]. Since silica fume has a lower surface area compared to the ash samples [20], dissolution of the SF would be lower than RSA or RHA in AAS systems. This in turn would lead to a lower silica modulus in AAS samples containing SF compared to those containing RSA or RHA; and a lower silica modulus would lead to a lower drying shrinkage of AAS samples. 

## 4. Conclusions

This research study investigated the impacts of Rice husk ash (RHA), rice straw ash (RSA), and silica fume (SF) on alkali activated slag (AAS) properties. A new method of application of these materials, in which RSA, RHA, and SF were treated with NaOH, was used in this study. The heat of hydration results indicated that AAS samples containing RSA, RHA, or SF generated more heat of hydration compared to the control sample. Furthermore, the induction period of AAS hydration was significantly shorted when RSA, RHA, or SF were added to the system; the impacts of treated materials were even more noticeable. Therefore, adding SF or RSA, particularly when they are treated, in AAS systems could accelerate the set time and early strength gain of the systems. It was found that addition of RSA, RHA, or SF in AAS mortar samples would decrease mortar workability.

It was shown that adding RHA and RSA as partial replacement of slag improves the compressive strength of AAS. Samples containing SF showed a lower compressive strength compared to those containing RHA or RSA. It was found that treating RSA and RHA significantly improved their performance in AAS systems. Results indicated that AAS samples containing treated RSA or RHA had a comparable compressive strength to those containing sodium silicate. It was also found that SF considerably reduced the drying shrinkage of AAS samples. However, treated RSA and RHA significantly increased the drying shrinkage of the AAS mortar samples.

## Figures and Tables

**Table 1:** Chemical composition of the materials used.

		SF	RHA	RSA	Slag
SiO_2_	%	98.5	93.8	87.0	31.9
Al_2_O_3_	%	0.2	0.1	0.4	11.6
Fe_2_O_3_	%	0.14	0.14	0.46	0.71
CaO	%	0.0	0.0	1.2	38.3
MgO	%	0.2	0.4	0.9	7.6
SO_3_	%	0.05	0.04	0.18	3.98
LOI	%	2.3	4.0	4.6	3.8
Na_2_O	%	0.10	0.15	0.60	0.16
K_2_O	%	0.60	0.62	1.60	0.28
P_2_O_5_	%	0.10	0.33	0.78	0.00

**Table 2:** Modulus ratios (SiO_2_/Na_2_O) and sodium oxide content of activator solutions.

Solution Type	(SiO_2_/Na_2_O)	Na_2_O %
NaOH (4 M)	0.00	8.68
NaOH + 5%RSA-T	0.28	8.68
NaOH + 10%RSA-T	0.56	8.68
NaOH + 5%RHA-T	0.30	8.68
NaOH + 10%RHA-T	0.60	8.68
NaOH + 5%SF-T	0.32	8.68
NaOH + 10%SF-T	0.63	8.68
12.5% Na-Si	0.29	8.38
20% Na-Si	0.58	8.52

**Table 3:** Flow of alkali activated slag mortars prepared with 4M NaOH.

Sample ID	Flow (%)	Sample ID	Flow (%)	Sample ID	Flow (%)
Slag	144	Slag	144	Slag	144
Slag + 5%RSA	136	Slag + 5%RHA	134	Slag + 5%SF	152
Slag + 10%RSA	132	Slag + 10%RHA	132	Slag + 10%SF	144
Slag + 5%RSA-T	110	Slag + 5%RHA-T	150	Slag + 5%SF-T	128
Slag + 10%RSA-T	90	Slag + 10%RHA-T	57	Slag + 10%SF-T	131

## Data Availability

Data is contained within the article.
